# Constructing an Adapted Cascade of Diabetes Care Using Inpatient Admissions Data: Cross-sectional Study

**DOI:** 10.2196/27486

**Published:** 2022-03-25

**Authors:** Irene Ryan, Cynthia Herrick, Mary F E Ebeling, Randi Foraker

**Affiliations:** 1 Institute for Public Health Washington University St Louis, MO United States; 2 Department of Medicine Washington University School of Medicine St. Louis, MO United States; 3 Department of Sociology Drexel University Philadelphia, PA United States; 4 Institute for Informatics Washington University in St. Louis, School of Medicine St. Louis, MO United States

**Keywords:** diabetes mellitus, cascade of care, EHR data, health care monitoring, inpatient care

## Abstract

**Background:**

The diabetes mellitus cascade of care has been constructed to evaluate diabetes care at a population level by determining the percentage of individuals diagnosed and linked to care as well as their reported glycemic control.

**Objective:**

We sought to adapt the cascade of care to an inpatient-only setting using the electronic health record (EHR) data of 81,633 patients with type 2 diabetes.

**Methods:**

In this adaptation, *linkage to care* was defined as prescription of diabetes medications within 3 months of discharge, and *control* was defined as hemoglobin A_1c_ (HbA_1c_) below individual target levels, as these are the most reliably captured items in the inpatient setting. We applied the cascade model to assess differences in demographics and percent loss at each stage of the cascade; we then conducted two-sample chi-square equality of proportions tests for each demographic. Based on findings in the previous literature, we hypothesized that women, Black patients, younger patients (<45 years old), uninsured patients, and patients living in an economically deprived area called the Promise Zone would be disproportionately unlinked and uncontrolled. We also predicted that patients who received inpatient glycemic care would be more likely to reach glycemic control.

**Results:**

We found that out of 81,633 patients, 28,716 (35.2%) were linked to care via medication prescription. Women and younger patients were slightly less likely to be linked to care than their male and older counterparts, while Black patients (n=19,141, 23.4% of diagnosed population vs n=6741, 23.5% of the linked population) were as proportionately part of the linked population as White patients (n=58,291, 71.4% of diagnosed population vs n=20,402, 71.0% of the linked population). Those living in underserved communities (ie, the Promise Zone) and uninsured patients were slightly overrepresented (n=6789, 8.3% of diagnosed population vs n=2773, 9.7% of the linked population) in the linked population as compared to patients living in wealthier zip codes and those who were insured. Similar patterns were observed among those more likely to reach glycemic control via HbA_1c_. However, conclusions are limited by the relatively large amount of missing glycemic data.

**Conclusions:**

We conclude that inpatient EHR data do not adequately capture the care cascade as defined in the outpatient setting. In particular, missing data in this setting may preclude assessment of glycemic control. Future work should integrate inpatient and outpatient data sources to complete the picture of diabetes care.

## Introduction

A total of 34 million patients in the United States are currently diagnosed with diabetes mellitus, which equates to roughly 10% of the population. Diabetes was the seventh leading cause of death in 2017. Type 2 diabetes is further complicated by comorbidities, such as high blood pressure, cholesterol, and cardiovascular disease (CVD) [1], and accounts for roughly US $327 billion per year in the US health care system [2].

Overall, new cases of diabetes have been decreasing over the last decade, even among younger patients, while disparities by both race and education level were noted in the US Centers for Disease Control and Prevention’s 2017 diabetes report. The report indicated that Hispanic, Black, and Native American and Alaska Native patients had a higher prevalence of diabetes compared to White patients. Additionally, diabetes was almost twice as prevalent among adults without a high school diploma in contrast to adults who at least graduated and pursued further degrees [3]. It has been shown that Black patients receiving Medicare are also less likely than those not covered by Medicare to report well-controlled blood sugar [4], and women diagnosed with diabetes face increased risk of cardiac and kidney comorbidities compared to men [5].

The cascade of diabetes care exists to examine the treatment path from diagnosis to linkage to care for diabetes patients through follow-up visits with their primary care provider, prescription of diabetes medications, and visits with diabetes or nutritional specialists. Diabetes *control* refers to adherence to quality-of-care metrics, including hemoglobin A_1c_ (HbA_1c_) measurements below individualized target levels, controlled systolic and diastolic blood pressure, assessment of lipids and urine microalbumin, and nonsmoking status.

Using National Health and Nutrition Examination Survey data from 2005 to 2016, previous research found that 70% of patients were linked to care, while just 20% of patients reached the composite treatment targets [6]. They reported that diabetes care, in terms of linkage to care and control, had not improved significantly from 2005-2006 to 2015-2016, nor were there improvements in disparities in linkage to care and disease control between sexes, races, and age groups. Younger patients and female patients were consistently less likely to be linked to care after their diagnosis. In addition, younger, female, non-White, and Hispanic patients were less likely to reach glycemic control, blood pressure, and cholesterol targets than their older, male, White, and non-Hispanic counterparts [7]. These disparities have been explored in previous studies compiled by the American Journal of Public Health [8] and have been tied to different behavioral factors, such as stress and substance abuse; psychological factors, such as depression; and clinical factors, such as quality of care and timely diagnosis and treatment.

According to the American Diabetes Association (ADA), roughly one-third of health care costs associated with diabetes are related to hospital inpatient care [9]. Basal-bolus insulin regimens for inpatient glycemic control have been shown to reduce hospital complications, particularly for postsurgical patients [10,11], with potentially improved glycemic control in subsequent follow-up periods as well [12]. While diabetes is seldom the primary focus of an inpatient admission, a hospitalization is nonetheless an opportunity for diabetes diagnosis and linkage to care, and this has not been thoroughly studied. Therefore, we sought to address this gap in the literature by examining a proposed inpatient cascade of diabetes care for patients with type 2 diabetes. This paper provides a construction and analysis of this framework by examining patients who were initially diagnosed in the inpatient setting and their linkage to care during their stay. Additionally, we assessed for disparities in care according to the demographic and socioeconomic characteristics of patients.

## Methods

### Study Population

Our study population was comprised of 93,433 patients with diabetes who were seen in the inpatient setting of a 15-hospital health care system in St. Louis, Missouri, from 2010 to 2019. We focused our study on individuals with or without complications of diabetes, and we excluded those diagnosed with diabetes during pregnancy (International Classification of Diseases, Tenth Revision [ICD-10] O24.xx, n=3875). Additionally, we excluded patients with type 1 diabetes (ICD-10 E10.xx, n=7925) from our study, given our objective to focus on those diagnosed with type 2 diabetes. We also focused on only those for which the diagnosis of type 2 diabetes appeared in the problem list for that admission. Our final study population included 81,633 patients.

### Constructing the Inpatient Cascade of Care

We consulted published guidelines from the ADA [9,10,13] with input from an endocrinologist (CH) to adapt the outpatient cascade of care [6] for the inpatient setting. Data were procured from the electronic health records (EHRs) of a large St. Louis, Missouri–area medical center. For the inpatient cascade, patients were considered diagnosed, linked to care, or controlled based on the following:

- Patients were considered diagnosed if they had a diagnosis of type 2 diabetes mellitus, with or without complications (ICD-10 E11.xx), and were admitted to the hospital system with their first recorded diabetes diagnosis in the problem list within a day of diagnosis.
- Patients were considered linked to care if they were prescribed insulin, noninsulin injectables, or oral anti-diabetes drugs (Multimedia Appendix 1) within 3 months of discharge.
- Patients were considered controlled if their recorded HbA_1c_ 6 months after discharge was between 7% and 8.5%. This definition was individualized per existing guidelines and the previously published outpatient cascade of care [6].
  Patients less than 65 years of age were considered controlled (HbA_1c_≤7%) if they had diabetes without complications or CVD, and were considered controlled (HbA_1c_≤8%) if they had complications or CVD.
  Patients 65 years of age or older were considered controlled (HbA_1c_≤7.5%) if they had diabetes without complications or CVD, and were considered controlled (HbA_1c_≤8.5%) if they had complications or CVD.

### Variables of Interest

Our population was mainly White, consistent with the population in the St. Louis metropolitan area. Thus, we categorized race into categories of White, Black, and other. We defined age groups according to the following cutoffs: 18 to 44 years, 45 to 64 years, and 65 years of age and older [6]. Patients with self-pay insurance or no recorded insurance were considered uninsured, and patients with insurance that did not include Medicare or Medicaid were considered privately insured.

We classified the zip code of residence for each patient according to whether or not they lived in the Promise Zone. The Promise Zone is an area in North St. Louis City and County designated in 2015 that is defined by a poverty rate or extremely low–income rate equal to or greater than 33% of the federal poverty level, where a federal local partnership has been established to improve education, economic activity, health, and wellness in the community [14].

To assess which HbA_1c_ control cutoff to use, we considered patients’ diabetes diagnoses, as well as any CVD diagnoses as previously described, and the patients’ ages. Our definition of CVD included myocardial infarction, heart failure, and angina (Multimedia Appendix 1). Finally, as a proxy to evaluate if and how diabetes was recognized in the inpatient setting (ie, inpatient glycemic care indicator), we assessed whether HbA_1c_ was measured, whether insulin lispro was administered within 24 hours of their admission, or whether a consultation with a diabetes or endocrinology specialist was called at any point during their admission.

### Analysis

We constructed descriptive statistics of the distributions of race, sex, and age at diagnosis, as well as insurance status, residence in the Promise Zone, and inpatient glycemic care indicators: HbA_1c_, insulin, and diabetes consultation in our data set. We then compared proportions within each category along the cascade of care to assess disparities using two-sample chi-square tests.

We explored geographic disparities in the data and compared the cascade of diabetes care for patients who lived in Promise Zone zip codes to those residing in the remaining zip codes in the patient catchment area.

### Hypotheses

We hypothesized that patients living in Promise Zone zip codes and those with self-pay insurance or on Medicaid would be less likely to be linked to care and to reach HbA_1c_ control. Additionally, we hypothesized that patients who had an inpatient glycemic care indicator would be more likely to be linked but less likely to be controlled, as those patients may be more severe cases to begin with to require inpatient insulin treatment. From findings in the previous literature, we hypothesized that women, Black patients, and younger patients would be disproportionately unlinked and uncontrolled [7].

### Ethical Considerations

This study was approved by the Washington University St. Louis Institutional Review Board (IRB) as an “exempt” project. The IRB ID for this project was 202007104.

## Results

### Overview

In our data set, 81,633 patients met the inclusion criteria. Our study population was 48.9% (n=39,880) female, 51.1% (n=41,748) male, and predominately White (n=58,291, 71.4%) and older (≥65 years of age: n=46,860, 57.4%; Table 1). A total of 35.5% (n=28,997) of patients were privately insured and 38.9% (n=31,742) received Medicare. A total of 17.5% (n=14,309) of patients resided in a Promise Zone zip code (Table 1), compared to 8% of the general St. Louis metropolitan population (data not shown).

**Table 1:** Demographic and clinical characteristics of our study population.

Variable			Participants (N=81,633), n (%)		
**Sex**					
	Female	39,880 (48.9)			
	Male	41,748 (51.1)			
	Missing	5 (<1.0)			
**Race**					
	White	58,291 (71.4)			
	Black	19,141 (23.4)			
	Other	2625 (3.2)			
	Missing	1576 (1.8)			
**Age at diagnosis (years)**					
	18-44	5887 (7.2)			
	45-64	28,886 (35.4)			
	≥65	46,860 (57.4)			
	Missing	4 (<1.0)			
Residence in Promise Zone			14,309 (17.5)		
**Insurance status**					
	Private	28,997 (35.5)			
	Medicare	31,742 (38.9)			
	Medicaid	14,096 (17.3)			
	Uninsured or self-pay	6798 (8.3)			
	Missing	4 (<1.0)			
**Inpatient glycemic care markers**					
	HbA_1c_^a^ measured within 24 hours	22,337 (27.4)			
	Insulin lispro administered within 24 hours	59,220 (72.5)			
	Diabetes or endocrinology specialist consultation during stay	4988 (6.1)			
	At least one glycemic care marker	63,222 (77.4)			

^a^HbA_1c_: hemoglobin A_1c_.

### The Inpatient Cascade of Care

**Figure 1:**
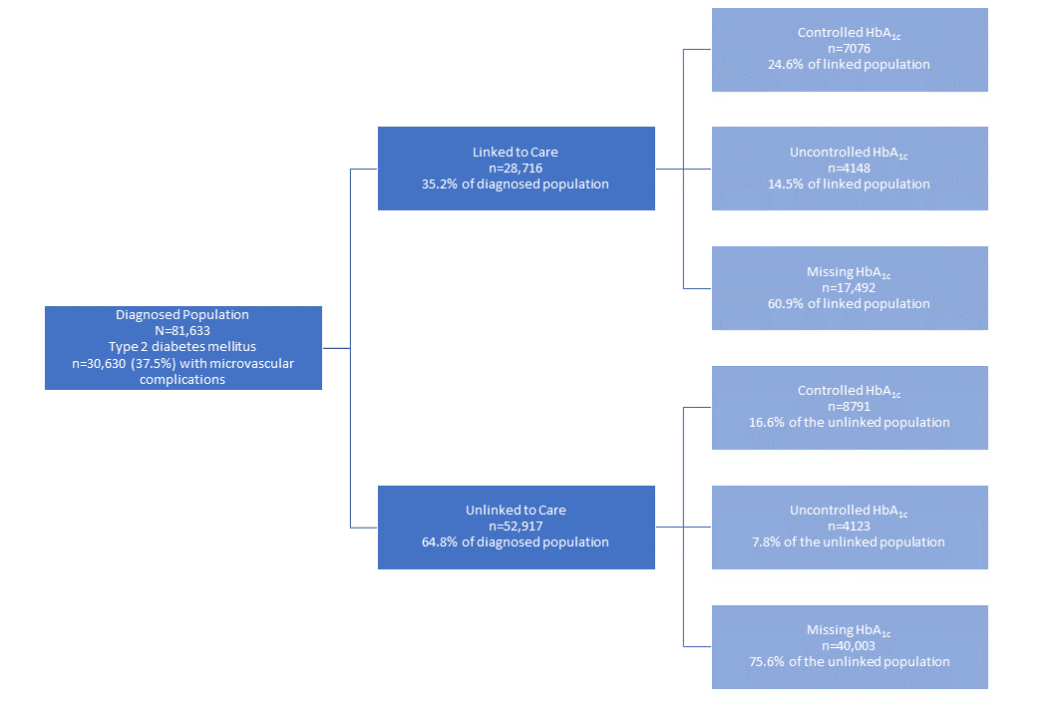
This flowchart illustrates the cascade of type 2 diabetes care with cohort sizes at each stage of the cascade, including information about missing glycemic data. Note, the control row of the flowchart indicates the percentage of the linked or unlinked cohorts, not the percentage of the original diagnosed cohort. HbA_1c_: hemoglobin A_1c_.

Out of 81,633 patients in our data set, 35.2% (n=28,716) met the linkage-to-care criteria for medication prescription within 3 months of discharge. Figure 1 shows the proportion of patients achieving each of the stages in the cascade of care, stratified by linked and unlinked to care, and then by in control, not in control, and missing a measure of HbA_1c_. A total of 70.4% of patients (n=57,495) had no HbA_1c_ values recorded in the inpatient setting 6 months after their initial admission. While this is a large proportion of missing values, we recognize that after an initial inpatient encounter, diabetes patients generally transition into outpatient settings for their continued care. Therefore, these values may not be recorded in inpatient EHR records.

In our data, females and younger patients were less likely to be linked to care compared to males and middle-aged patients. Women made up 48.9% (n=39,880) of the diagnosed population, but 48.9% (n=13,814) of the linked population (<1% change, *P*=.03). Younger patients made up 7.2% (n=5887) of the entire diagnosed population, but were just 6.8% (n=1963) of the linked population (<1% change, *P*=.03). We did not find that patient race varied between the diagnosed, linked, and unlinked populations. Patients living in the Promise Zone (n=14,309, 17.5% of diagnosed population vs n=5366, 18.7% of linked population; *P*<.001) and uninsured patients (n=6798, 8.3% vs n=2773, 9.7%; *P*<.001) were more likely to be linked to care, while those with private insurance were less likely to be linked to care (n=28,997, 35.5% vs n=9611, 33.5%; *P*<.001).

Differences in HbA_1c_ control among individuals linked to care must be interpreted with caution in the setting of missing data. Among those linked to care, patients 65 years of age and older and men were less likely to be uncontrolled compared to younger patients and women. Black patients were overrepresented in the uncontrolled group, but they were also less likely to be missing a recorded HbA_1c_ value at 6 months as measured in the inpatient setting. While patients in the Promise Zone were more likely to be linked to care, approximately one-third (n=1612, 30.0% of linked patients residing in the Promise Zone) of those patients reached HbA_1c_ control. Finally, individuals with self-pay insurance and those on Medicaid were less likely to be controlled but were also less likely to be missing HbA_1c_ data over the course of the 6-month follow-up window.

**Table 2:** Cascade of care by population covariates.

Variable		Diagnosed (N=81,633), n (%)	Linked to care (n=28,716), n (%)	Unlinked to care (n=52,917), n (%)	Linked to care, n (%)^a^		
					Controlled HbA_1c_^b^ (n=7076)	Uncontrolled HbA_1c_ (n=4148)	Missing HbA_1c_ (n=17,492)
**Sex**							
	Female	39,880 (48.9)	13,814 (48.1)*	26,066 (49.3)	3592 (50.8)*	2102 (50.7)*	8120 (46.4)*
	Male	41,748 (51.1)	14,902 (51.9)*	26,846 (50.7)	3484 (49.2)*	2046 (49.3)*	9372 (53.6)*
	Missing	5 (<1.0)	0 (0)	5 (<1.0)	0 (0)	0 (0)	0 (0)
**Race**							
	White	58,291 (71.4)	20,402 (71.0)	37,889 (71.6)	4789 (67.7)*	2584 (62.3)*	13,029 (74.5)*
	Black	19,141 (23.4)	6741 (23.5)	12,400 (23.4)	2012 (28.4)*	1418 (34.2)*	3311 (18.9)*
	Other	2625 (3.2)	1001 (3.5)*	1624 (3.1)	246 (3.0)	109 (2.6)	679 (3.9)*
	Missing	1576 (1.8)	572 (1.9)	1004 (1.9)	74 (<1.0)	32 (<1.0)	473 (2.7)*
**Age at diagnosis (years)**							
	18-44	5887 (7.2)	1963 (6.8)*	2924 (7.4)*	349 (4.9)*	516 (12.4)*	1098 (6.3)*
	45-64	28,886 (35.4)	10,397 (36.2)*	18,489 (34.9)	2376 (33.6)*	2130 (51.4)*	5891 (33.7)*
	≥65	46,860 (57.4)	16,356 (57.0)	30,504 (57.6)	4351 (61.5)*	1502 (36.2)*	10,503 (60.0)*
Residence in Promise Zone		14,309 (17.5)	5366 (18.7)*	8943 (16.9)*	1612 (22.9)*	847 (28.5)*	2661 (15.2)*
**Insurance status**							
	Private	28,997 (35.5)	9611 (33.5)*	19,386 (36.6)*	1937 (27.4)*	1140 (38.4)*	6145 (35.1)*
	Medicare	31,742 (38.9)	11,487 (40.0)*	20,255 (38.2)*	3132 (44.3)*	731 (24.6)*	7152 (40.9)
	Medicaid	14,096 (17.3)	4845 (16.9)	9251 (17.5)	1261 (17.8)	583 (19.6)*	2813 (16.1)*
	Self-pay	6798 (8.3)	2773 (9.7)*	4025 (7.6)*	746 (10.5)*	515 (17.3)*	1382 (7.9)*
**Inpatient glycemic care markers**							
	HbA_1c_ measured within 24 hours	22,337 (27.4)	8255 (28.7)*	14,082 (26.6)*	1979 (28.0)*	1390 (33.5)*	4886 (27.9)
	Insulin lispro administered within 24 hours	59,220 (72.5)	23,003 (80.1)*	36,217 (68.4)*	5589 (79.0)*	3649 (88.0)*	13,765 (78.7)*
	Diabetes or endocrinology specialist during stay	4988 (6.1)	2335 (8.1)*	2654 (5.0)*	737 (10.4)*	687 (16.5)*	910 (5.2)*
	At least one inpatient glycemic care marker	63,222 (77.4)	24,159 (84.1)*	39,064 (73.8)*	5937 (83.9)	3800 (91.6)*	14,421 (82.4)*

^a^Proportions in the glycemic control columns denote the proportion of linked patients, not of the entire diagnosed population.

^b^HbA_1c_: hemoglobin A_1c_.

*P<.05 between proportions from one stage of the cascade to the next.

Patients with inpatient glycemic care markers were more likely to be linked to care. For example, 72.5% (n=59,220) of the diagnosed population were administered insulin lispro within 24 hours, and those patients made up 80.1% (n=23,003) of the linked cohort versus 68.4% (n=36,217) of the unlinked cohort (*P*<.001). Additionally, 77.4% (n=63,222) of patients in the diagnosed population had at least one inpatient glycemic care indicator, and these patients were also overrepresented in the linked population (linked: n=24,159, 84.1% vs unlinked: n=39,064, 73.8%; *P*<.001). However, individuals with at least one glycemic care indicator were overrepresented in the uncontrolled population (uncontrolled: n=3800, 91.6% vs controlled: n=5937, 83.9%; *P*<.001).

## Discussion

### Principal Findings

The analysis in this study is a first step in using inpatient EHR data to explore the diabetes cascade of care in an inpatient setting. Among a population of individuals with type 2 diabetes diagnoses newly noted during hospital admission, we were able to determine how many were linked to care using a definition of diabetes medication prescription within 3 months of discharge. We were able to determine indicators of inpatient glycemic management (ie, HbA_1c_ checked, insulin started, or diabetes service consulted) and identified that individuals with one or more of these indicators were significantly more likely to be linked to care. Our findings highlight the importance of recognizing diabetes during a hospital stay in establishing appropriate follow-up.

We observed that a significant proportion of individuals treated with insulin lispro during a hospital stay did not meet criteria for linkage to care, identifying a potential opportunity for intervention. In addition, very few patients were seen by a diabetes or endocrinology specialist in the hospital; however, this is not unexpected given that diabetes consultations, when available at a particular hospital, are typically reserved for only the most severely uncontrolled patients. We also examined HbA_1c_ values at 6 months postdischarge; however, conclusions about control were limited by significant *missingness* in the data, and these associations should be explored with more complete data in the future.

Our analysis highlights three important points: (1) a possible “leaky pipeline,” or patients that drop out of the cascade of care after discharge from the hospital; (2) the importance of recognizing and acting on diabetes in the inpatient setting to mitigate this leaky pipeline; and (3) the difficulty of using inpatient data alone for the definition of a diabetes cascade of care.

### Linkage to Care, Leaky Pipeline, and Inpatient Glycemic Care Markers

First, we noted that only 35% of the population was linked to care by our definition of receiving medication for diabetes within 3 months of discharge. This likely overestimates loss to follow-up; however, because 19% of patients in the unlinked group were noted to have a controlled HbA_1c_ at 6 months postdischarge, these patients were likely linked to outpatient care. Nonetheless, even if those who were linked to care and those who were unlinked but controlled were considered together, they would comprise 54% of the population. This proportion is much lower than the 77% who met one of the inpatient glycemic care indicators in the inpatient setting.

We observed that patients with at least one of the inpatient glycemic care markers were much more likely to be linked to care than those without, underscoring the importance of recognizing and acting on diabetes during a hospital stay. Additionally, and not surprisingly, at 6 months postdischarge, a larger proportion of individuals who were linked to care had controlled HbA_1c_ as compared to those not linked to care, and a smaller proportion of those who were linked to care had missing HbA_1c_ data as compared to those not linked to care. We hypothesized that while patients with inpatient glycemic care markers were more likely to be linked to care, they would also be less likely to reach their HbA_1c_ target at 6 months, possibly representing diabetes that is more difficult to control.

Counter to our hypothesis, patients with no insurance were more likely to be linked to care, and patients with private insurance were less likely to be linked. Meanwhile, patients living in the Promise Zone were more likely to be linked to care as compared to those not residing in the Promise Zone. These findings were unexpected but could reflect the type of follow-up data available within our hospital system. Privately insured individuals and those living in zip codes with higher economic opportunity may be more likely to follow up with a physician outside of our system, whereas uninsured patients and those living in the Promise Zone may be more likely to be readmitted to our system as an inpatient during the follow-up period.

### Glycemic Control Assessment and Data Missingness

Data *missingness* refers to the prevalence of data not captured in the inpatient EHR. Black patients, younger patients, patients with no insurance, or those receiving Medicaid were overrepresented in the uncontrolled population as compared with the overall diagnosed population; however, these groups were also less likely to be missing HbA_1c_ data than their comparators, so it is difficult to draw firm conclusions with this information. The lower proportion of missing HbA_1c_ data among these populations may be related to these populations being seen in the inpatient setting more often; thus, their data were better captured.

### Difficulty Defining Diabetes Cascade of Care With Inpatient Data Alone

Overall, our analysis demonstrates that defining a cascade of diabetes care using inpatient data alone is limited by the fragmentation in the health care ecosystem. Given that diabetes is managed almost exclusively in the outpatient setting, the utility of an inpatient diabetes cascade of care would be primarily to (1) determine appropriate management of hyperglycemia in the inpatient setting for individuals with diabetes and (2) identify newly diagnosed diabetes and action by the inpatient team to prescribe appropriate therapy and connect the patient to an outpatient provider upon discharge. Our analysis highlights a challenge commonly encountered in the United States, in that inpatient data may not be linked to outpatient data, even within a network of affiliated outpatient practices in a hospital system [15,16]. Interoperability of EHR systems is critical to optimize this type of analysis in the future.

### The “Ideal” Inpatient Cascade of Diabetes Care

An optimal investigation of a diabetes cascade of care would focus on diabetes first noted in the inpatient setting and identifying individuals first noted in a hospital stay to have glucose values outside of the normal range. The next steps would be to determine what proportion of these individuals subsequently had an HbA_1c_ evaluation, and then to quantify how many of those diagnosed with diabetes via an HbA_1c_ value equal to or more than 6.5% were initiated on diabetes medication and referred to a primary care provider for follow-up. Such an investigation would also include detailed glucose data during the hospital stay to determine what proportion of individuals with blood sugars greater than 180 mg/dL in the inpatient setting were started on insulin during the hospital stay and how many had a diabetes consultation in the hospital. The cascade would then follow individuals in the outpatient setting to determine how many ultimately achieved glycemic control, blood pressure, cholesterol, and smoking targets, which are associated with CVD risk among patients with diabetes. The benefit of using fully integrated inpatient and outpatient EHR data for this type of analysis lies in the potential for longitudinal follow-up and the opportunity to identify individuals most severely affected by complicated diabetes who are hospitalized.

### System-Level Challenges in Transition of Care From Inpatient to Outpatient Settings

The lack of interoperability between inpatient and outpatient EHR systems creates significant opportunities for loss to follow-up in clinical care. Hospital discharge summaries may not reach the primary care physician, and they may not specifically address diabetes if this was not the reason for admission [17,18]. Moreover, enhanced diabetes education in the inpatient setting with specific transition instructions provided to the patient and primary care physician improved HbA_1c_ over a 1-year follow-up [19]. However, inpatient diabetes self-management education is not a reimbursed service, and outpatient access to certified diabetes education is limited [20]. Diabetes-specific structured communication between inpatient and outpatient providers is essential to improve diabetes follow-up along the cascade of care.

### Limitations

Significant limitations exist in the study, as outlined in the previous section, in part due to fragmentation. This is evidenced in the high number of missing HbA_1c_ measures due to the limitations of inpatient EHR data that we had access to for the analysis. Additionally, we pulled the first inpatient admission of diabetes mellitus based on the problem list for each patient, which means they could have been diagnosed earlier in the outpatient or primary care setting and received linkage to care for their diabetes in another setting not captured in our analysis.

### Conclusions

An inpatient encounter may be an opportunity for incidental diabetes diagnosis, treatment, and linkage to care. However, a cascade of diabetes care using inpatient data alone is insufficient and difficult to align with the outpatient cascade of diabetes care [6] because of differences in care delivery and guidelines between the two settings. Additionally, we noted that while there were statistically significant differences between demographic variables of sex, race, age, insurance, and socioeconomic status indicated by residence in the Promise Zone and patients’ linkage to care and glycemic control, those relationships may not be clinically significant. We recommend further study using integrated EHR data from inpatient and outpatient settings to define a cascade of care across the continuum of care to better define the utility of the inpatient setting in capturing and linking individuals with diabetes to appropriate outpatient care.

## Appendix

Multimedia Appendix 1 Variable definitions using administrative codes.

## Abbreviations

ADA: American Diabetes Association
CDTR: Center for Diabetes Translation Research
CVD: cardiovascular disease
EHR: electronic health record
HbA_1c_: hemoglobin A_1c_
ICD-10: International Classification of Diseases, Tenth Revision
IRB: Institutional Review Board
NICHD: National Institute of Child Health and Human Development

## References

[1] (2020). Standards of Medical Care in Diabetes—2020. Diabetes Care. Volume 43, Supplement 1.

[2] Statistics about diabetes. American Diabetes Association.

[3] Centers for Disease Control and Prevention (2020). National Diabetes Statistics Report, 2020.

[4] Addressing health disparities in diabetes. Centers for Disease Control and Prevention.

[5] Kautzky-Willer A, Harreiter J, Pacini G (2016). Sex and gender differences in risk, pathophysiology and complications of type 2 diabetes mellitus. Endocr Rev.

[6] Kazemian P, Shebl FM, McCann N, Walensky RP, Wexler DJ (2019). Evaluation of the cascade of diabetes care in the United States, 2005-2016. JAMA Intern Med.

[7] CMS, OMH, NORC (2017). Racial and Ethnic Disparities in Diabetes Prevalence, Self-Management, and Health Outcomes Among Medicare Beneficiaries. CMS OMH Data Highlight No. 6.

[8] Black SA (2002). Diabetes, diversity, and disparity: What do we do with the evidence?. Am J Public Health.

[9] American Diabetes Association (2018). Economic costs of diabetes in the US in 2017. Diabetes Care.

[10] Umpierrez GE, Smiley D, Zisman A, Prieto LM, Palacio A, Ceron M, Puig A, Mejia R (2007). Randomized study of basal-bolus insulin therapy in the inpatient management of patients with type 2 diabetes (RABBIT 2 trial). Diabetes Care.

[11] Umpierrez GE, Smiley D, Jacobs S, Peng L, Temponi A, Mulligan P, Umpierrez D, Newton C, Olson D, Rizzo M (2011). Randomized study of basal-bolus insulin therapy in the inpatient management of patients with type 2 diabetes undergoing general surgery (RABBIT 2 surgery). Diabetes Care.

[12] Hellman R (2014). An individualized inpatient diabetes education and hospital transition program for poorly controlled hospitalized patients with diabetes. Endocr Pract.

[13] American Diabetes Association (2019). 15. Diabetes care in the hospital: Standards of Medical Care in Diabetes-2019. Diabetes Care.

[14] (2019). St. Louis Promise Zone Progress Report.

[15] Kruse CS, Stein A, Thomas H, Kaur H (2018). The use of electronic health records to support population health: A systematic review of the literature. J Med Syst.

[16] Quinn M, Forman J, Harrod M, Winter S, Fowler KE, Krein SL, Gupta A, Saint S, Singh H, Chopra V (2019). Electronic health records, communication, and data sharing: Challenges and opportunities for improving the diagnostic process. Diagnosis (Berl).

[17] Robelia PM, Kashiwagi DT, Jenkins SM, Newman JS, Sorita A (2017). Information transfer and the hospital discharge summary: National primary care provider perspectives of challenges and opportunities. J Am Board Fam Med.

[18] Kripalani S, LeFevre F, Phillips CO, Williams MV, Basaviah P, Baker DW (2007). Deficits in communication and information transfer between hospital-based and primary care physicians: Implications for patient safety and continuity of care. JAMA.

[19] Wexler DJ, Beauharnais CC, Regan S, Nathan DM, Cagliero E, Larkin ME (2012). Impact of inpatient diabetes management, education, and improved discharge transition on glycemic control 12 months after discharge. Diabetes Res Clin Pract.

[20] Rutledge SA, Masalovich S, Blacher RJ, Saunders MM (2017). Diabetes self-management education programs in nonmetropolitan counties - United States, 2016. MMWR Surveill Summ.

