# Supplementary material for: Constructing an Adapted Cascade of Diabetes Care Using Inpatient Admissions Data: Cross-sectional Study
Source: JMIR Diabetes. 2022 Mar 25;7(1):e27486. doi: 10.2196/27486 (PMC8994153; doi:10.2196/27486)
Supplement: Multimedia Appendix 1 [file diabetes_v7i1e27486_app1.docx]

*Multimedia Appendix 1*

*Variable definitions utilizing administrative codes*

| *Diabetes Definitions used to define final population* | ICD-10 codes |  |
| --- | --- | --- |
| **Inclusion** |  |  |
| Type 2 diabetes |  |  |
| **Without complications** | E11.9 |  |
| **With microvascular complications** | E11.xx | (00,21,22,29,31,32,35,40,42,43,51,61,62,64,65,69,80) |
| **Exclusion** |  |  |
| Gestational diabetes | O24.xx |  |
| Type I diabetes | E10.xx |  |
|  |  |  |
|  |  |  |
| *Medication names used to define linkage to care* |  |  |
| Insulin | aspart, glulisine, lispro, degludec, detemir, glargine, regular, NPH, humulin, novolin, humalog, novolog |  |
| Oral or non-insulin injectable | liraglutide, semaglutide, dulaglutide, exanitide, lixisenatide, sitagliptin, linagliptin, saxagliptin, alogliptin, dapagliflozin, canagliflozin, empagliflozin, ertugliflozin, metformin, glipizide, glyburide, glimepiride, pioglitazone, rosiglitazone, nateglinide, repaglinide, pramlintide |  |
